# Mitogenomic phylogenetic analyses of *Leptogorgia virgulata* and *Leptogorgia hebes* (Anthozoa: Octocorallia) from the Gulf of Mexico provides insight on Gorgoniidae divergence between Pacific and Atlantic lineages

**DOI:** 10.1002/ece3.5847

**Published:** 2019-11-21

**Authors:** Samantha Silvestri, Diego F. Figueroa, David Hicks, Nicole J. Figueroa

**Affiliations:** ^1^ School of Earth, Environmental, and Marine Sciences University of Texas Rio Grande Valley Brownsville TX USA

**Keywords:** divergence, evolution, mitochondrial genome, MutS, octocoral, phylogeography, sea whip

## Abstract

The use of genetics in recent years has brought to light the need to reevaluate the classification of many gorgonian octocorals. This study focuses on two *Leptogorgia* species—*Leptogorgia virgulata* and *Leptogorgia hebes*—from the northwestern Gulf of Mexico (GOM). We target complete mitochondrial genomes and *mtMutS* sequences, and integrate this data with previous genetic research of gorgonian corals to resolve phylogenetic relationships and estimate divergence times. This study contributes the first complete mitochondrial genomes for *L. ptogorgia virgulata* and *L. hebes*. Our resulting phylogenies stress the need to redefine the taxonomy of the genus *Leptogorgia* in its entirety. The fossil‐calibrated divergence times for Eastern Pacific and Western Atlantic *Leptogorgia* species based on complete mitochondrial genomes shows that the use of multiple genes results in estimates of more recent speciation events than previous research based on single genes. These more recent divergence times are in agreement with geologic data pertaining to the formation of the Isthmus of Panama.

## Graphical abstract

This study contributes the first complete mitochondrial genomes for *Leptogorgia virgulata* and *Leptogorgia hebes*. The fossil‐calibrated divergence times based on complete mitochondrial genomes shows that the use of multiple genes, as opposed to a single gene, results in estimates of more recent speciation events than previously published for Eastern Pacific and Western Atlantic *Leptogorgia* species. These more recent divergence times are in agreement with geologic data pertaining to the formation of the Isthmus of Panama.

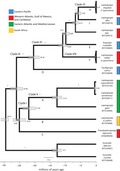

## 1 INTRODUCTION

There are 54 valid species in the genus *Leptogorgia* belonging to the family Gorgoniidae (Milne‐Edwards & Haime, 1857). They are classified as soft corals due to their lack of a protective calcium carbonate exoskeleton. Instead, for support and protection, they rely on small, calcitic structures called sclerites (O'Neal & Pawlik, 2002), from which their white, translucent polyps protrude, and they range in color from yellow to orange to red to purple (White & Strychar, 2010). *Leptogorgia* and other octocorals provide habitat heterogeneity and therefore allow for large aggregations of diverse fauna (Quattrini et al., 2014). Greater habitat complexity has been shown to be significantly correlated with higher red snapper abundance, an economically important fish species in the Gulf of Mexico and Western Atlantic Ocean (Szedlmayer, 2007). There are nine species of *Leptogorgia* in the Gulf of Mexico, including *Leptogorgia hebes* and *Leptogorgia virgulata* (Cairns & Bayer, 2009). They are found at depths ranging from 2 to 309 m, with the depth range of *L. hebes* ranging from 9 to 37 m and that of *L. virgulata* from 3 to 82 m (Cairns & Bayer, 2009; Williamson, Strychar, & Withers, 2011). *Leptogorgia hebes* and *L. virgulata* reach reproductive maturity within 2 years, and both are broadcast spawners, releasing eggs, and sperm into the water column (Beasley, Dardeau, & Schroeder, 2003; Gotelli, 1991). The larvae in *L. virgulata* can spend 3–20 days in the water column before settlement (Gotelli, 1991). The duration of the larval stage for *L. hebes* is unknown. Both *L. hebes* and *L. virgulata* have been successful at colonizing artificial structures in the Gulf of Mexico, including jetties within the subtidal zone (Williamson et al., 2011). A strong holdfast and a rigid, yet flexible skeleton, allows these two species to colonize habitats with swift currents and wave action such as that found in jetties (Williamson et al., 2011). These life history characteristics of *L. hebes* and *L. virgulata*, which include relatively fast maturation, broadcast spawning, long survival of larval stages, and adaptations for successful establishment in high energy environments, demonstrate the high potential for dispersal and colonization of new regions of these species.

Old, incomplete, or damaged records in addition to a lack of easily identifiable morphological traits among species make gorgonians particularly difficult to classify (Sánchez, 2007). For example, the genus *Leptogorgia* was initially split into two genera—*Leptogorgia* and *Lophogorgia*—by Milne‐Edwards and Haime (1857). Species in the *Leptogorgia* genus are described as having disk‐spindles in the outer coenenchyme, while *Lophogorgia* species have spindles more closely resembling flat rods and were described mostly in the Eastern Pacific, Western Atlantic, Caribbean, and along the eastern and southern coasts of Africa (Bayer, 1961). However, in 1988 these morphological distinctions were questioned and the two genera were united into one as *Leptogorgia* (Grasshoff, 1988). In 2017, Poliseno et al. conducted a phylogenetic study of *Leptogorgia*, using specimens from a wide geographical area, including the eastern and Western Atlantic, the Eastern Pacific, and the Mediterranean. They reconstructed two phylogenies, one based on complete mitochondrial genomes and the other based on a partial fragment of the mitochondrial *MutS* gene (*mtMutS*). While their phylogeny based on complete mitochondrial genomes only has eleven species of the family Gorgoniidae, including six species of *Leptogorgia*, the one based on the single *mtMutS* gene includes 109 species, providing greater taxonomic resolution. In their study, Poliseno et al. (2017) also estimate divergence times with a fossil calibration based on the oldest known fossil of *Eunicella*, dating back to 28.4 Ma (Kocurko & Kocurko, 1992) using the partial *mtMutS* gene. Based on their results, Poliseno et al. (2017) call for a global taxonomic revision of the present‐day *Leptogorgia* genus. They conclude from the *mtMutS* phylogeny that the genus *Lophogorgia* should be resurrected for all South African *Leptogorgia* species, which form an old clade within the Gorgoniidae, sister to *Leptogorgia* species from the eastern coast of Africa and the Mediterranean. They show that these Eastern Atlantic *Leptogorgia* species diverged from Western Atlantic species in the late Cretaceous, about 65 Ma, while the divergence between the Western Atlantic and Eastern Pacific species occurs more recently, between 28 and 23 Ma. These observations not only raise the question of taxonomic placement and nomenclature for Eastern Pacific and Western Atlantic species, but also suggest a divergence time between these lineages that dates back to the very early stages of emergence of the Isthmus of Panama (Bacon et al., 2015). This scenario is unexpected since *Leptogorgia* are shallow water species and significant exchange of seawater between the two basins likely occurred until ~10–15 Ma when the final stages of the closure of the Central American Seaway (CAS) started, with shallow water still connecting these two oceans until 3.5–4.2 Ma with the final rise of the Isthmus of Panama (e.g., Bacon et al., 2015; Montes et al., 2015; O'Dea et al., 2016).

Our study focuses on two species of *Leptogorgia* from the Gulf of Mexico, *L. hebes* and *L. virgulata*. We have two main goals. The first is to determine the taxonomic position of *L. hebes* (formerly classified in the genus *Lophogorgia* by Bayer, 1961) and of *L. virgulata*. The analyses by Poliseno et al. (2017) did not include complete mitochondrial genomes for these two species and their phylogeny based on the partial *mtMutS* gene leaves the phylogenetic position of both *L. hebes* and *L. virgulata* weakly supported. Therefore, in our study, we analyze both complete mitochondrial genomes and the *mtMutS* gene. Although mitochondrial genomes have been shown to be problematic for phylogenetic reconstruction of scleractinian corals due to the presence of substitution saturation and long branch attraction (i.e., Kitahara et al., 2014), it is only an issue within the Hexacorallia and it does not affect the Octocorallia, such as the gorgonian corals in our study (Figueroa & Baco, 2015). Complete mitochondrial genomes have been demonstrated to provide robust and well‐supported phylogenies for Octocorallia (e.g., Figueroa & Baco, 2014; Figueroa & Baco, 2015; Kayal, Roure, Philippe, Collins, & Lavrov, 2013; Poliseno et al., 2017), while the use of single mitochondrial genes has been demonstrated to result in incongruent largely unresolved trees across a wide range of taxa (Havird & Santos, 2014; Knaus, Cronn, Liston, Pilgrim, & Schwartz, 2011; Luo et al., 2011; Nadimi, Daubois, & Hijri, 2016; Pacheco et al., 2011; Rohland et al., 2007; Urantowka, Kroczak, & Mackiewicz, 2017; Wang et al., 2017; Willerslev et al., 2009). Therefore, we expect that the taxonomic position of *L. hebes* and *L. virgulata* will be fully resolved by reconstructing their phylogeny using mitochondrial genomes.

Our second goal is to estimate divergence times of Eastern Pacific and Western Atlantic *Leptogorgia* species. Since previous research has shown that fossil‐calibrated phylogenetic reconstruction based on single mitochondrial genes results in an overestimation of divergence times (Duchêne, Archer, Vilstrup, Caballero, & Morin, 2011; McCormack, Heled, Delaney, Peterson, & Knowles, 2011), we will base our estimates of diversification times between Eastern Pacific and Western Atlantic lineages of *Leptogorgia* by targeting complete mitochondrial genomes. We reconstruct a fossil‐calibrated phylogenetic tree for *Leptogorgia* species based con complete mitochondrial genomes and using *Eunicella* as an outgroup. We use a fossil calibration point of 28.4 Ma based on the stratigraphy and dating of the Red Bluff Formation in Mississippi where the oldest fossils of *Eunicella* have been recovered (Cushing, Boswell, & Hosman, 1964; Demchuk & Gary, 2009; Kocurko & Kocurko, 1992; Prothero, Ivany, & Nesbitt, 2003; Tew, 1992). Among Octocorallia, skeletal diversity, such as morphology of sclerites, is a key character for taxonomic identification (Goffredo & Dubinsky, 2016). Sclerites with a balloon club shape are a distinguishing characteristic that is unique to the genus *Eunicella* (Goffredo & Dubinsky, 2016; Kocurko & Kocurko, 1992). Fossil sclerites with balloon club shape have been found in the Red Bluff Formation in Mississippi and have been clearly attributed to *Eunicella* (Kocurko & Kocurko, 1992). Stratigraphy of the Red Bluff Formation and dating of this layer within the Oligocene (23–34 Ma) has been intensely studied (i.e., Cushing et al., 1964; Demchuk & Gary, 2009; Hosman, 1996; Prothero et al., 2003; Tew, 1992).

The timeline proposed by Poliseno et al. (2017) for the divergence between Eastern Pacific and Western Atlantic *Leptogorgia* species coincides with evidence that a land bridge between North and South America began to emerge between 23 and 25 Ma when the Panama Arc collided with South America (Bacon et al., 2015). However, despite this initial emergence and given the life history characteristics of shallow water *Leptogorgia* species such as *L. hebes* and *L. virgulata* that enhance dispersal and colonization (Beasley et al., 2003; Cairns & Bayer, 2009; Gotelli, 1988, 1991; Williamson et al., 2011), gene flow is likely to have continued between the Western Atlantic and Eastern Pacific until full closure of the Central American Seaway (Bacon et al., 2015; Cowman & Bellwood, 2013; Lessios, 2008; Thacker, 2017).. Therefore, we hypothesize that the divergence times of Eastern Pacific and Western Atlantic *Leptogorgia* lineages to be younger than previously suggested (Poliseno et al., 2017) with the majority of speciation events occurring after 10 Ma when significant seawater exchange between the Pacific and Atlantic Ocean ceased (i.e., Bacon et al., 2015; Montes et al., 2015; O'Dea et al., 2016).

## 2 MATERIALS AND METHODS

### 2.1 Study sites and sample collection

Six sites in the Gulf of Mexico off the coast of the United States in South Padre Island, Texas, were sampled for 24 *Leptogorgia* specimens (seven *L. hebes* and 17 *L. virgulata*) by divers collecting coral fragments between June 2014 and July 2017 (Table 1). Once collected, samples were preserved in ethanol and stored at 0°C. Voucher specimens are deposited and curated at the University of Texas Rio Grande Valley's Coastal Studies Laboratory and are available upon request under GenBank accession numbers MK0301586–MK0301592 for specimens of *L. virgulata* and MN052675–MN052677 for specimens of *L. hebes*.

**Table 1:** *Leptogorgia* sp. found at all sites and site types with respective dates, coordinates, and depths

Location	Date	Lat	Lon	Depth (m)	# of samples/species collected
Port Isabel Reef	6/3/2014	25.9684	−97.0669	22	1 *L. hebes;* 1 *L. virgulata*
Port Mansfield Liberty Ship	12/12/2014	26.4296	−97.0241	24	1 *L. hebes;* 1 *L. virgulata*
Jack up Rigs/East Bank	7/7/2016	26.1021	−96.9377	32	1 *L. virgulata*
Port Mansfield Liberty Ship	9/2/2016	26.4296	−97.0241	24	1 *L. virgulata*
Port Isabel Reef	9/15/2016	25.9684	−97.0669	22	*1 L. hebes;* *1 L. virgulata*
Port Mansfield Liberty Ship	3/20/2017	26.4296	−97.0241	24	1 *L. hebes*
MU 726 A	6/8/2017	27.8146	−96.7622	24	4 *L. virgulata*
Texas Clipper	7/8/2017	26.1903	−96.8614	15–41	2 *L. virgulata*
SPI Jetty	7/30/2017	26.0674	−97.1504	5	3 *L. hebes;* 6 *L. virgulata*

### 2.2 DNA Extraction and PCR

Three to five individual polyps were picked off from each coral sample, depending on the size and quality of preservation of the coral fragment. Polyps were visually inspected under a stereo microscope and picked off the coral stalk using forceps. Forceps were sterilized in between each sample using 100% bleach and 100% ethanol. If individual polyps were difficult to distinguish, an ~0.5 cm long piece was broken off of the coral fragment. The PureLink Genomic DNA Mini Kit (Thermo Fisher Scientific) was used to extract DNA from each sample following the manufacturer's standard protocol. Prior to extraction, coral polyps were rehydrated for 1–2 hr in molecular grade water and then digested for at least 5 hr. The final DNA product was eluted two times for maximum yield. The elution buffer was heated to 55°C prior to use, and 60 µl of were used for both elutions. The concentration of the extracted DNA was measured using a Qubit fluorometer (Life Technologies Inc.).

Polymerase Chain Reaction (PCR) amplification was performed on 0.1–5.0 ng template DNA from 24 samples in order to target the mtMutS gene with forward primer ND42599F (GCCATTATGGTTAACTATTAC; France & Hoover, 2002) and reverse primer Mut3458R (TSGAGCAAAAGCCACTCC; Sanchez, McFadden, France, & Lasker, 2003). The PCR mix consisted of the following in 25 μl total volume: 16.05 µl nuclease free water, 2.5 Invitrogen's 10X PCR Rxn Buffer, 1.25 µl Invitrogen's 50 mM MgCl, 2.0 µl of 10 mM dNTP, 1.0 µl of 10 mM forward primer (ND42599F), 1.0 µl of 10 mM reverse primer (Mut3458R), 0.2 µl Thermo Fisher's Invitrogen Platinum TAQ DNA Polymerase, and 1.0 µl DNA. Samples were then amplified in an Eppendorf Mastercycler pro thermocycler using the following parameters: 94°C for 2 min, 35 cycles at 94°C for 1 min, 50°C for 1 min, 72°C for 1 min, and a final step at 72°C for 5 min. The resulting product was visualized by gel electrophoresis on an ultraviolet light transilluminator to assess DNA length and quality. Once all samples yielded successful amplification, the final PCR product was then purified with the Invitrogen PureLink PCR Purification Kit, following the manufacturer's procedure. The primers and purified PCR products were then sent to Eurofins Genomics for sequencing of forward and reverse strands.

The genomic DNA extraction from each specimen was visualized after gel electrophoresis with an ultraviolet light transilluminator. Genomic DNA of high molecular weight with minimal degradation was identified by looking for high concentrations above 5,000 bp, with minimal streaking below this size. Based on these observations, the mitochondrial genome of ten specimens with the highest quality of genomic DNA was targeted using next generation sequencing technology. The genomic DNA extraction of these ten specimens was used to prepare an indexed library following standard procedures with the Nextera X2 kit. These 10 libraries, along with 86 libraries from other projects, were multiplexed and sequenced on a 100 bp paired‐end lane of Illumina HiSeq 2500 at Harvard's Biopolymers facility. The sequences were de‐multiplexed according to their indices.

### 2.3 Sequence assembly and alignment

For each specimen, the sequences for the forward and reverse strands were assembled with the software CLC Workbench 7.9.1 (CLC Bio) using the settings: minimum aligned read length = 500 bp, alignment stringency = high, conflicts = ambiguity nucleotides, trim sequence ends and trim using quality scores limit = 0.05. A cutoff was used were only bases with Phred scores of 20 or more were kept. A consensus sequence was generated from each assembly. Qiagen's CLC Workbench 7.9.1 was used to align the *mtMutS* sequences. The *mtMutS* sequences were aligned using Qiagen's CLC Main Workbench 7 software and include 24 sequences from this study, the 114 sequences examined in Poliseno et al. (2017) and 43 novel sequences available in GenBank for a total of 182 sequences (Table 2). The alignment was visually inspected for errors and inconsistencies. The final *mtMutS* alignment was 766 bp in length.

**Table 2:** All 182 *Leptogorgia mtMutS* sequences incorporated into the *mtMutS* phylogeny with corresponding GenBank accession numbers

Species	Accession #	Species	Accession #	Species	Accession #	Species	Accession #
*Leptogorgia virgulata* a	MN159153	*Leptogorgia alba* b	KX721205	*Leptogorgia obscura* b	KX721210	*Leptogorgia taboguilla* c	LT221102
*Leptogorgia virgulata* a	MN159154	*Leptogorgia alba* b	KX721203	*Leptogorgia piccola* c	AY268444	*Leptogorgia tricorata* c	LT221111
*Leptogorgia virgulata* a	MN159155	*Leptogorgia alba* b	KX721202	*Leptogorgia pulcherrima* c	KY683795	*Leptogorgia tricorata* c	LT221109
*Leptogorgia virgulata* a	MN159156	*Leptogorgia alba* b	KX721201	*Leptogorgia pulcherrima* c	KY683794	*Leptogorgia viminalis* c	KY683797
*Leptogorgia virgulata* a	MN159157	*Leptogorgia alba* b	KX721195	*Leptogorgia pulcherrima* c	KY683793	*Leptogorgia viminalis* c	KY683796
*Leptogorgia virgulata* a	MN159158	*Leptogorgia alba* b	KY559410	*Leptogorgia pulcherrima* c	AY268443	*Leptogorgia violacea* c	AY268448
*Leptogorgia virgulata* a	MN159159	*Leptogorgia alba* b	KX767314	*Leptogorgia pumila* b	KX767312	*Leptogorgia violetta* c	AY268446
*Leptogorgia virgulata* a	MN159160	*Leptogorgia alba* c	HG917036	*Leptogorgia pumila* c	LT221116	*Leptogorgia virgulata* c	AY268458
*Leptogorgia virgulata* a	MN159161	*Leptogorgia alba* c	HG917035	*Leptogorgia punicea* c	AY268449	*Leptogorgia virgulata* c	AY126418
*Leptogorgia virgulata* a	MN159162	*Leptogorgia alba* c	HG917034	*Leptogorgia ramulus* c	AY268451	*Pacifigorgia bayeri* c	HG917044
*Leptogorgia virgulata* a	MN159163	*Leptogorgia alba* c	AY268452	*Leptogorgia ramulus* b	KX767322	*Pacifigorgia cairnsi* c	KY559409
*Leptogorgia virgulata* a	MN159164	*Leptogorgia alba* c	LT221108	*Leptogorgia regis* c	LT221101	*Pacifigorgia cairnsi* c	HG917041
*Leptogorgia virgulata* a	MN159165	*Leptogorgia alba* c	LT221113	*Leptogorgia regis* c	LT221100	*Pacifigorgia catedralensis* c	HG917019
*Leptogorgia virgulata* a	MN159166	*Leptogorgia barnardi* c	KY236043	*Leptogorgia regis* c	LT221099	*Pacifigorgia cf. cairnsi* c	HG917046
*Leptogorgia virgulata* a	MN159167	*Leptogorgia capverdensis* c	KY553145	*Leptogorgia regis* c	LT221098	*Pacifigorgia cf. cairnsi* c	HG917021
*Leptogorgia virgulata* a	MN159168	*Leptogorgia cf.palma* c	KY559406	*Leptogorgia rigida* c	GQ342496	*Pacifigorgia exilis* b	KX351871
*Leptogorgia virgulata* a	MN159169	*Leptogorgia cf. gilchristi* c	KY236042	*Leptogorgia rubra* b	KX767323	*Pacifigorgia firma* b	KX351872
*Leptogorgia hebes* a	MN159170	*Leptogorgia cf. palma* c	KY236030	*Leptogorgia sarmentosa* c	KY559411	*Pacifigorgia firma* c	HG917022
*Leptogorgia hebes* a	MN159171	*Leptogorgia cf. palma* c	KY236031	*Leptogorgia* sp.^b^	KX767315	*Pacifigorgia irene* b	KX351873
*Leptogorgia hebes* a	MN159172	*Leptogorgia chilensis* c	AY268460	*Leptogorgia* sp.^b^	KX721204	*Pacifigorgia irene* c	HG917024
*Leptogorgia hebes* a	MN159173	*Leptogorgia chilensis* c	JN866554	*Leptogorgia* sp.^b^	KY559412	*Pacifigorgia machalilla* b	KX351876
*Leptogorgia hebes* a	MN159174	*Leptogorgia cofrini* c	HG917037	*Leptogorgia* sp. *2* c	KY236033	*Pacifigorgia machalilla* b	KX351874
*Leptogorgia hebes* a	MN159175	*Leptogorgia cofrini* c	HG917040	*Leptogorgia* sp. *2* c	KY236032	*Pacifigorgia machalilla* b	KX351875
*Leptogorgia hebes* a	MN159176	*Leptogorgia cofrini* c	HG917039	*Leptogorgia* sp.^c^	KY683791	*Pacifigorgia media* c	GQ342497
*Acanthogorgia* sp^c^	AY268461	*Leptogorgia cofrini* c	HG917038	*Leptogorgia* sp.^c^	LT221114	*Pacifigorgia rubicunda* c	HG917027
*Antillogorgia acerosa* b	JX152763	*Leptogorgia cortesi* c	LT221105	*Leptogorgia* sp.^c^	LT221115	*Pacifigorgia sculpta* b	KX351877
*Antillogorgia sp. 1* b	JX152764	*Leptogorgia cuspidata* b	KX767318	*Leptogorgia* sp.^c^	LT221106	*Pacifigorgia senta* c	LT221107
*Antillogorgia sp. 2* b	JX152765	*Leptogorgia cuspidata* c	AY268450	*Leptogorgia* sp. *3* c	KY236035	*Pacifigorgia smithsoniana* c	HG917023
*Eugorgia ampla* b	KX767316	*Leptogorgia cuspidata* c	HG917047	*Leptogorgia* sp. *3* c	KY236034	*Pacifigorgia stenobrochis* c	HG917018
*Eugorgia daniana* c	HG917048	*Leptogorgia dichotoma* c	AY268445	*Leptogorgia* sp. *4* c	KY236037	*Pacifigorgia stenobrochis* c	AY126420
*Eugorgia daniana* c	LT221110	*Leptogorgia diffusa* b	KX767319	*Leptogorgia* sp. *4* c	KY236036	*Phyllogorgia dilatata* c	AY126428
*Eugorgia multifida* c	GQ342494	*Leptogorgia diffusa* b	KX721209	*Leptogorgia* sp. *5* c	KY236039	*Pseudopterogorgia acerosa* c	AY126421
*Eugorgia mutabilis* c	KY559405	*Leptogorgia flexilis* b	KX767326	*Leptogorgia* sp. *5* c	KY236038	*Pseudopterogorgia americana* c	AY126423
*Eugorgia mutabilis* c	LT221112	*Leptogorgia flexilis* b	KX767329	*Leptogorgia* sp. *6* c	KY236041	*Pseudopterogorgia australiensis* c	AY268442
*Eugorgia rubens* c	JN866557	*Leptogorgia flexilis* b	KX767328	*Leptogorgia* sp. *6* c	KY236040	*Pseudopterogorgia bipinnata* c	DQ640646
*Eugorgia siedenburgae* c	LT221097	*Leptogorgia flexilis* b	KX767327	*Leptogorgia* sp.^b^	KX721200	*Pseudopterogorgia elisabethae* c	AY126422
*Eugorgia siedenburgae* c	LT221096	*Leptogorgia flexilis* b	KX767325	*Leptogorgia* sp.^b^	KX721199	*Pseudopterogorgia fredericki* b	JX152766
*Eugorgia siedenburgae* c	LT221094	*Leptogorgia flexilis* b	KX767473	*Leptogorgia* sp.^b^	KX721197	*Pseudopterogorgia rubrotincta* b	JX152768
*Eunicea mammosa* b	JX152767	*Leptogorgia flexilis* b	KX767474	*Leptogorgia* sp.^b^	KX721196	*Eunicella cavolinii* c	JQ397290
*Eunicella albicans* c	KY559407	*Leptogorgia gaini* c	KY559404	*Leptogorgia* sp.^b^	KX721198	*Eunicella singularis* c	JQ397296
*Eunicella cavolinii* c	KY559408	*Leptogorgia gracilis* c	AY268454	*Leptogorgia styx* c	AY268453	*Eunicella* sp.^c^	JQ397310
*Gorgonia flabellum* c	AY126427	*Leptogorgia hebes* c	AY268459	*Leptogorgia sylvanae* c	KY683792	*Eunicella verrucosa* c	JQ397300
*Gorgonia mariae* c	AY126426	*Leptogorgia labiata* c	AY268447	*Leptogorgia sylvanae* c	KY683798		
*Gorgonia ventalina* c	AY126425	*Leptogorgia mariarosae* b	KX721193	*Leptogorgia taboguilla* c	LT221103		
*Leptogorgia alba* b	KX767324	*Leptogorgia obscura* b	KX767321	*Leptogorgia taboguilla* c	LT221093		
*Leptogorgia alba* b	KX721206	*Leptogorgia obscura* b	KX767320	*Leptogorgia taboguilla* c	LT221104		

a Sequences from this study.

b New sequences from GenBank.

c Sequences used by Poliseno et al. (2017).

The Illumina sequence reads were assembled using the software CLC Genomics Workbench 11. Default settings were used with reads mapped back to contigs (mismatch cost = 2, insertion cost = 3, deletion cost = 3, length fraction = 0.5, similarity fraction = 0.8). The sequences obtained from the assemblies included the full mitochondrial genome for each specimen with an average read coverage of over 100 and a minimum coverage of 35. The assembled genomes were annotated using Qiagen CLC Genomics Workbench 11 software using previously published *Leptogorgia mt* genomes as references (Table 3). The ten mitochondrial genomes obtained were analyzed along with eleven mitochondrial genomes available in GenBank (Table 3). Individual genes and RNAs were extracted and aligned separately using MUSCLE v3.8 (Edgar, 2004) with default parameters. The alignments were visually inspected for consistency. The resulting alignments were then concatenated for phylogenetic analyses and deposited in the online database http://figshare.com under https://doi.org/10.6084/m9.figshare.10052030.

**Table 3:** All 21 gorgonian mitochondrial genomes and their corresponding GenBank accession number

Species	Size (bp)	GenBank Accession #
*Leptogorgia virgulata* a	18,845	MK301586
*Leptogorgia virgulata* a	18,845	MK301587
*Leptogorgia virgulata* a	18,824	MK301588
*Leptogorgia virgulata* a	18,845	MK301589
*Leptogorgia virgulata* a	18,845	MK301590
*Leptogorgia virgulata* a	18,824	MK301591
*Leptogorgia virgulata* a	18,845	MK301592
*Leptogorgia hebes* a	19,247	MN052675
*Leptogorgia hebes* a	19,247	MN052676
*Leptogorgia hebes* a	19,247	MN052677
*Pseudopterogorgia bipinnata*	18,733	DQ640646
*Leptogorgia capverdensis*	18,722	KY553145
*Leptogorgia gaini*	19,682	KY559404
*Eugorgia mutabilis*	19,157	KY559405
*Leptogorgia cf. palma*	18,731	KY559406
*Eunicella albicans*	19,175	KY559407
*Eunicella cavolinii*	19,316	KY559408
*Pacifigorgia cairnsi*	19,156	KY559409
*Leptogorgia alba*	18,848	KY559410
*Leptogorgia sarmentosa*	18,722	KY559411
*Leptogorgia* sp.	18,849	KY559412

a The 10 novel *mt* genomes sequenced in this study.

### 2.4 Phylogenetic analyses

Both *mtMutS* and complete *mt* genome alignments were used in phylogenetic analyses using maximum likelihood (ML) and Bayesian methods. The model of evolution and partitioning scheme was determined by PartitionFinder v1.1.1 (Lanfear, Calcott, Kainer, Mayer, & Stamatakis, 2014) using linked branches and the Akaike information criterion (AIC). The RAxML v8.0.0 program (Stamatakis, 2017) was used to conduct the ML analyses and Mr. Bayes 3.1 (Ronquist & Huelsenbeck, 2003) was used for the Bayesian analyses. Data blocks were created for *mtMutS* based on codon position (Table 4). PartitionFinder selected GTR + G as the best evolutionary model for three partitions: (a) mtMutS^1^; (b) mtMutS^2^; and (c) mtMutS^3^.

**Table 4:** Data block definitions for partition analysis

Region	Codon positions		
	1	2	3
(A)			
Atp6	1–708	2–708	3–708
Atp8	709–924	710–924	711–924
Cox1	925–2,550	926–2,550	927–2,550
Cox2	2,551–3,312	2,552–3,312	2,553–3,312
Cox3	3,313–4,098	3,314–4,098	3,315–4,098
Cytb	4,099–5,273	4,100–5,273	4,101–5,273
MutS	5,274–8,231	5,275–8,231	5,276–8,231
Nad1	8,232–9,203	8,233–9,203	8,234–9,203
Nad2	9,204–10,361	9,205–10,361	9,206–10,361
Nad3	10,362–10,734	10,363–10,734	10,364–10,734
Nad4	10,735–12,183	10,736–12,183	10,737–12,183
Nad4L	12,184–12,477	12,185–12,477	12,186–12,477
Nad5	12,478–14,320	12,479–14,320	12,480–14,320
Nad6	14,321–14,878	14,322–14,878	14,323–14,878
rRNA (12s)	14,879–15,807		
rRNA (16s)	15,808–17,999		
(B)			
MutS	1–766	2–766	3–766

Note

(A) Mitochondrial genome concatenated alignment including 14 protein‐coding genes and 2 RNAS. (B) *mtMutS* alignment.

For the mitochondrial genome analyses, data blocks were created based on codon positions for all 14 protein‐coding genes (*Cox1, Nad1, CytB, Nad6, Nad3, Nad4L,mtMutS, Nad2, Nad5, Nad4, Cox3, Atp6, Atp8,* and *Cox2*) and two ribosomal RNAs (Table 4). For the ML analysis, PartitionFinder selected General Time Reversible plus Gamma (GTR + G) as the best evolutionary model for 11 partition subsets and GTR + I+G for three subsets (Table 5). For the Bayesian analysis, the data were partitioned into 16 subsets. PartitionFinder selected GTR + I as the best model for two subsets, F81 for one subset, GTR + G for four subsets, GTR + I+G for two subsets, GTR for one subset, HKY for two subsets, HKY + G for two subsets, and HKY + I+G for one subset (Table 5).

**Table 5:** Partition scheme for the concatenated mitochondrial genome alignment for ML and Bayesian analyses

Subset	Best model	# of Sites	Maximum likelihood partitions
1	GTR + G	808	Nad2^1^, Atp6^1^, Nad6^1^
2	GTR + I+G	2,333	Nad6^2^, Nad4^2^, Cytb^2^, Nad5^2^, Atp6^2^, Nad4L^2^, Nad12
3	GTR + G	1,423	Cox1^3^, Cytb^3^, Cox2^3^, Atp6^3^
4	GTR + I+G	3,193	Atp8^1^, 16s rRNA, 12s rRNA
5	GTR + G	582	Nad2^2^, Atp8^2^, Nad3^2^
6	GTR + G	782	Nad2^3^, Nad1^3^, Atp8^3^
7	GTR + G	1,605	Cox2^1^, Nad4L^1^, Nad3^1^, Cox3^1^, Cox1^1^, Nad1^1^
8	GTR + G	1,058	Cox2^2^, Cox3^2^, Cox1^2^
9	GTR + G	546	Nad6^3^, Nad4L^3^, Cox3^3^
10	GTR + I+G	1,490	Nad5^1^, Cytb^1^, Nad4^1^
11	GTR + G	1,110	MutS^1^, Nad3^3^
12	GTR + G	986	MutS^2^
13	GTR + G	1,600	Nad5^3^, MutS^3^
14	GTR + G	483	Nad4^3^

Subset	Best model	# of Sites	Bayesian partitions
1	GTR + I	808	Nad6^1^, Atp6^1^, Nad2^1^
2	F81	720	Nad2^2^, Nad4L^2^, Atp6^2^
3	GTR + G	1,788	Atp8^3^, Cox1^3^, Cox2^3^, Atp6^3^, Nad1^3^, Nad4L^3^, Cox3^3^
4	HKY + I+G	3,193	Atp8^1^, 16s rRNA, 12s rRNA
5	HKY	196	Nad3^2^, Atp8^2^
6	GTR + I	1,351	Cox1^1^, Nad1^1^, Cox3^1^, Nad4L^1^, Nad3^1^
7	GTR + I	1,382	Cox1^2^, Nad1^2^, Cox3^2^, Cox2^2^
8	HKY	254	Cox2^1^
9	GTR + I+G	1,490	Cytb^1^, Nad4^1^, Nad5^1^
10	GTR + I+G	1,675	Cytb^2^, Nad5^2^, Nad4^2^, Nad6^2^
11	HKY + G	1,563	Nad6^3^, MutS^3^, Cytb^3^
12	HKY + G	1,110	MutS^1^, Nad3^3^
13	GTR + G	986	MutS^2^
14	GTR	386	Nad2^3^
15	GTR + G	483	Nad4^3^
16	GTR + G	614	Nad5^3^

Note

Superscript numbers indicate codon position 1, 2, or 3.

The best maximum likelihood tree was reconstructed with RAxML for both, the *mtMutS* alignment and the *mt* genome alignment, using bootstrap values from 10,000 replicates. Note that in RAxML partitions cannot be analyzed with different evolutionary models and one model must be used for all partitions. Therefore, the mt genome alignment was analyzed under a GTR + G model given that PartitionFinder selected this as the best model for 11 of the 14 partitions. Phylogenetic trees were also reconstructed for both by Bayesian methods. Using Mr. Bayes, four chains were carried out for 1,100,000 generations, sampling every 200th generation. After inspecting the trace files generated by the Bayesian Markov Chain Monte Carlo (MCMC) runs, the initial 100,000 of sampled generations were omitted prior to building the consensus tree. Both ML and Bayesian phylogenies were rooted with sequences of species of Eunicella downloaded from Genbank (Tables 2 and 3).

Divergence time estimates were performed by Bayesian analyses using full mitochondrial genomes only, with the software BEAST 2.3.2 (Bouckaert et al., 2019). The alignment was partitioned as specified above for the Bayesian phylogenetic reconstruction (Table 3). An uncorrelated log‐normal relaxed clock model was used along with the calibrated yule speciation model. The tree was calibrated based on the earliest fossil evidence for *Eunicella* (Kocurko & Kocurko, 1992) with a date of origination set to 28.4 Ma (mean = 1 and standard deviation = 1). One chain was carried out for 10,000,000 generations, sampling every 1,000th generation. After inspecting the trace files generated by the Bayesian Markov Chain Monte Carlo (MCMC) runs, the initial 25% of sampled generations were omitted prior to building the tree. Mean divergence times were summarized with TreeAnnotator.

## 3 RESULTS

### 3.1 Mitochondrial *MutS* phylogeny

**Figure 1:**
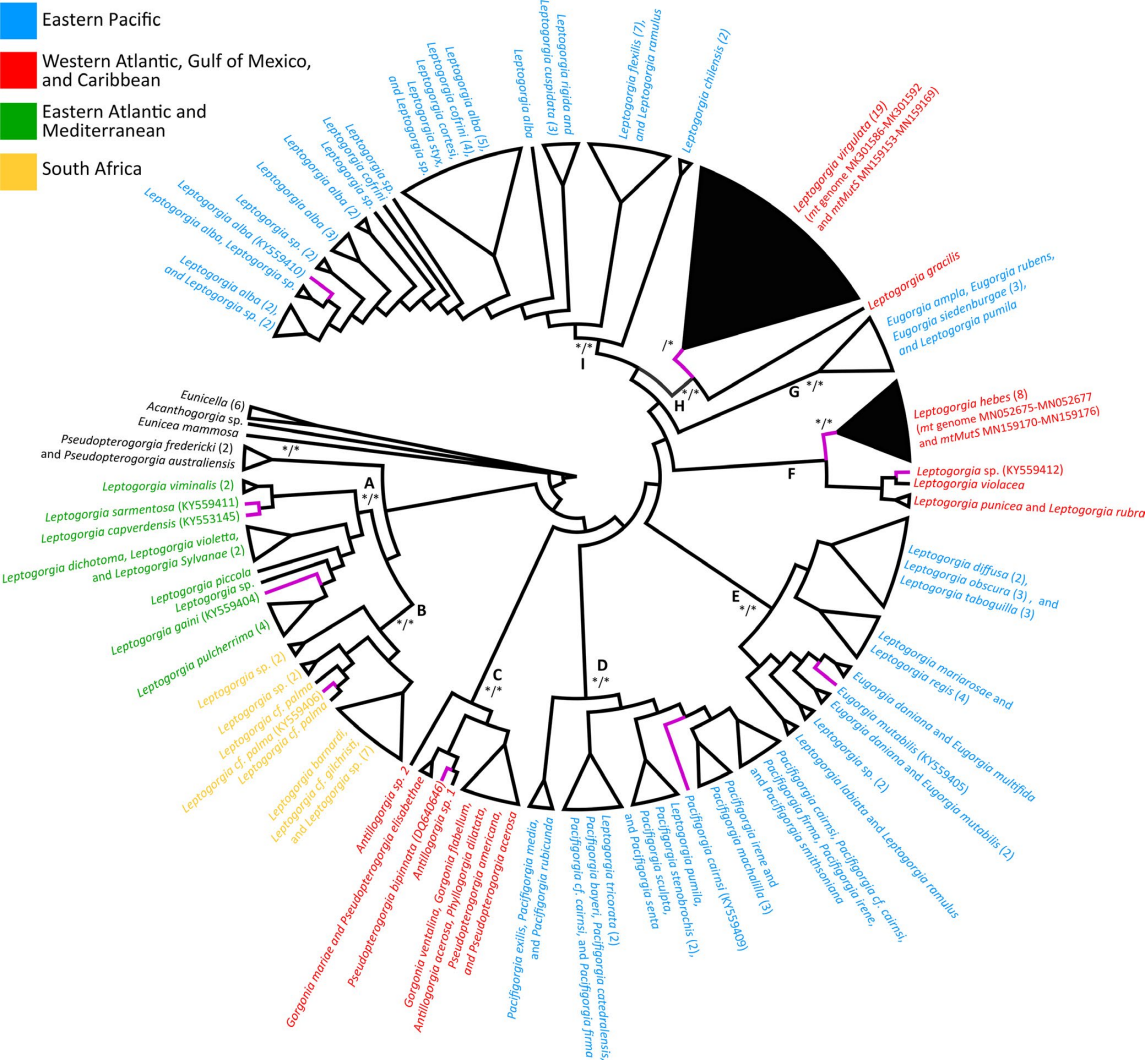
Maximum likelihood phylogenetic reconstruction of the genus *Leptogorgia* based on *mtMutS*. Major clades by geographic regions labeled A–I. Support values shown only for clades A–I and denoted by an * on branch, indicating strong support (>70 bootstrap/>95 posterior probability)

The 17 sequences of *mtMutS* of *L. virgulata* are identical, while the seven sequences of *L. hebes* range from 99.74% to 100% identity. The phylogenetic reconstruction based on *mtMutS* included the 17 sequences of *L. virgulata* and the seven sequences of *L. hebes* generated by this study (Figure 1). These 24 sequences were combined with 158 additional *Leptogorgia mtMutS* sequences and two of *Eunicella* (outgroup), downloaded from GenBank. The topology between the Bayesian and maximum likelihood analyses is relatively similar. There are nine major clades (A–I) that are strongly supported (>70 bootstrap and >95 posterior probability) except for Clade *F* (<50 bootstrap and <50 posterior probability), each corresponding to taxa from a particular geographic region (Figure 1). Clade A corresponds to species found in the Eastern Atlantic and Mediterranean. Clade A is sister to Clade B, which corresponds to species found in South Africa. Clade A and B, along with *Pseudopterogorgia fredericki* and *Pseudopterogorgia australiensis* form a clade that is weakly supported (51 bootstrap and 85 posterior probability) and sister to all other species (Figure 1). This sister clade with the remaining species is moderately supported (77 bootstrap and 86 posterior probability) and contains clades C–I. Clade C (100 bootstrap and posterior probability) consists of 11 different species representing multiple genera (*Pseudopterogorgia, Antillogorgia, Gorgonia,* and *Phyllogorgia*). All species in Clade C are found in the Caribbean (Figure 1). Clade C is sister to the remaining species which form a strongly supported group (97 bootstrap and 86 posterior probability) containing clades D–I. Clade D (100 bootstrap and posterior probability) is made up of species of *Pacifigorgia* along with a few species of *Leptogorgia*, all of which are from the Eastern Pacific (Figure 1). Clade D is sister to a strongly supported clade (100 bootstrap and 100 posterior probability) that consist of the remaining species within clades E–I. Clade E (89 bootstrap and 100 posterior probability) consists of several species of *Leptogorgia* and *Eugorgia* all from the Eastern Pacific (Figure 1). Clade E is sister to a strongly supported group (91 bootstrap and 100 posterior probability) containing clades F–I. Clade F does not have statistical support (<50 bootstrap and <50 posterior probability). Within clade F are *Leptogorgia violacea*, *L. punicea*, and *L. rubra* along with a well‐supported clade (90 bootstrap and 99 posterior probability) containing all specimens of *L. hebes* (Figure 1). All species in clade F are from the Western Atlantic and Gulf of Mexico. Clade F is sister to a clade with no statistical support (<50 bootstrap and <50 posterior probability) containing the remaining species within clades G‐I. Clade G (100 bootstrap and posterior probability) contains several species of *Eugorgia* along with *Leptogorgia pumila*, all from the Eastern Pacific (Figure 1). Clade G is sister to a group that is not statistically supported (<50 bootstrap and <50 posterior probability) and contains clades H and I. Clade H (100 bootstrap and posterior probability) consists of species from the Western Atlantic and Gulf of Mexico, *Leptogorgia gracilis* and *L. virgulata* (Figure 1). Specimens of *L. virgulata* form a clade that is moderately supported in the ML tree (64 bootstrap) and strongly supported in the Bayesian tree (100 posterior probability). This *L. virgulata* clade is sister to *L. gracilis*. Clade H is sister to Clade I (100 bootstrap and posterior probability) which contains numerous species of *Leptogorgia* from the Eastern Pacific (Figure 1).

### 3.2 Mitochondrial genomes

**Figure 2:**
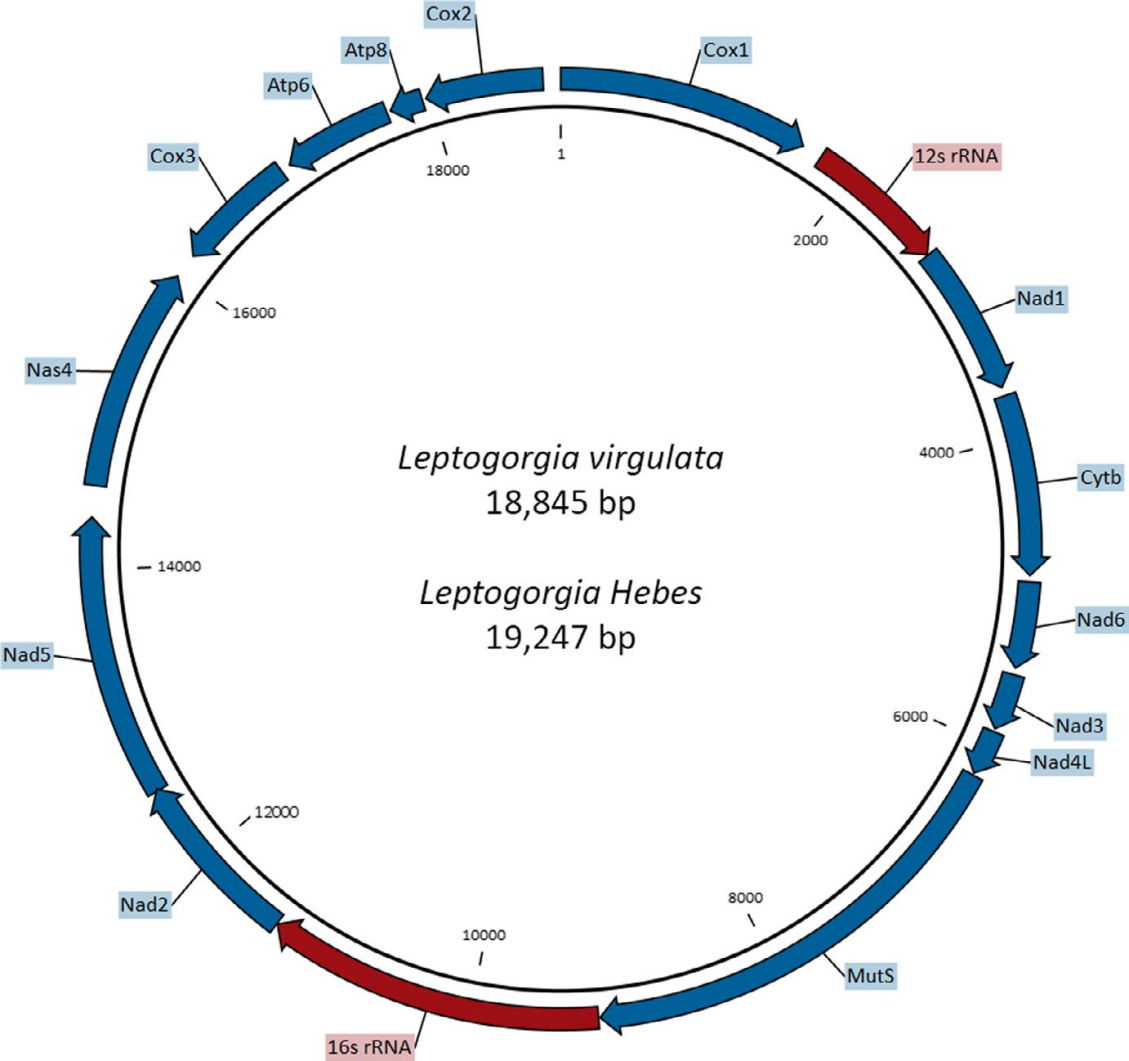
Complete mitochondrial genomes for *Leptogorgia virgulata* and *Leptogorgia hebes* including all 14 protein‐coding genes, shown in blue, and rRNAs, shown in red

A total of ten new *Leptogorgia* mitochondrial genomes were obtained—seven *L. virgulata mt* genomes and three *L. hebes mt* genomes. The *L. virgulata mt* genomes range in length from 18,824 to 18,845, and all *L. hebes mt* genomes are 19,247 bp. The *L. virgulata mt* genomes range from 99.87% to 100% identity while those for *L. hebes* range from 99.5% to 99.98% identity. All ten *mt* genomes consist of 14 protein‐coding genes (*Cox1, Nad1, CytB, Nad6, Nad3, Nad4L, mtMutS, Nad2, Nad5, Nad4, Cox3, Atp6, Atp8,* and *Cox2*, in respective order) and two ribosomal RNAs (Figure 2). Both species have what is presumed to be the ancestral gene order found in octocorals (Brugler & France, 2008; Figueroa & Baco, 2014, 2015; Medina, Collins, Takaoka, Kuehl, & Boore, 2006; Park et al., 2012; Uda et al., 2011).

### 3.3 Mitogenomic phylogeny

**Figure 3:**
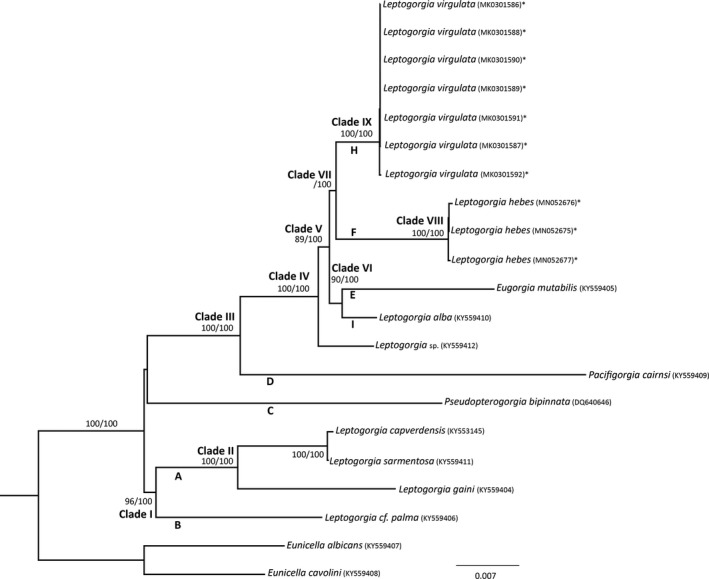
Maximum likelihood phylogenetic reconstruction of the family Gorgoniidae using complete mitogenomes. Major clades labeled I–IX. Branch labels show support values (bootstrap/posterior probability). * on species names indicates mitochondrial genomes generated by this study. Branches labeled A–H correspond to clades defined in the phylogeny based on *mtMutS*

The phylogenetic reconstruction based on full mitochondrial genomes included 7 *mt* genomes of *L. virgulata* and 3 *mt* genomes of *L. hebes* generated by this study. These *mt* genomes were combined with 11 additional *mt* genomes from the family Gorgoniidae and two *mt* genomes of *Eunicella* (outgroup), downloaded from GenBank (Table 3). Maximum likelihood and Bayesian analyses resulted in similar topology (Figure 3). There are nine well‐supported clades (clades I–IX) that roughly match those identified in the *mtMuS* phylogeny (Figure 3). Clade I (96 bootstrap and 100 posterior probability) is made up of *Leptogorgia palma* (*mtMuS* clade B) as sister to clade II (100 bootstrap and posterior probability, *mtMuS* clade A) which contains *Leptogorgia capverdensis* and *Leptogorgia sarmentosa*. Clade I is sister to all other *Leptogorgia*, but this sister clade is weakly supported (61 bootstrap and 85 posterior probability) and contains *Pseudopterogorgia bipinnata* (*mtMuS* clade C) as sister to clade III. Clade III (100 bootstrap and posterior probability) contains *Pacifigorgia cairnsi* (*mtMuS* clade D) as sister to clade IV (100 bootstrap and posterior probability). Clade IV contains *Leptogorgia* sp. (KY559412) as sister to clade V (89 bootstrap and 100 posterior probability). Clade V consists of clade VI (90 bootstrap and 100 posterior probability) as sister to clade VII (65 bootstrap and 100 posterior probability). Clade VI has *Leptogorgia alba* (*mtMuS* clade I) as sister to *Eugorgia mutabilis* (*mtMuS* clade E). Clade VII contains clade VIII (*mtMuS* clade F) as sister to clade IX (*mtMuS* clade H). Clade VIII (100 bootstrap and 100 posterior probability) consists of three specimens of *L. hebes*. Clade IX (100 bootstrap and posterior probability) consists of seven specimens of *L. virgulata*. Within the *L. hebes* clade, two individuals (accession #s MN052676 and MN052675) form a strongly supported clade (97 bootstrap and 81 posterior probability). The *L. virgulata* clade also has two individuals (accession #s MK0301589 and MK0301591) forming an internal clade, strongly supported by maximum likelihood only (96 bootstrap).

### 3.4 Mitogenomic divergence time estimation

**Figure 4:**
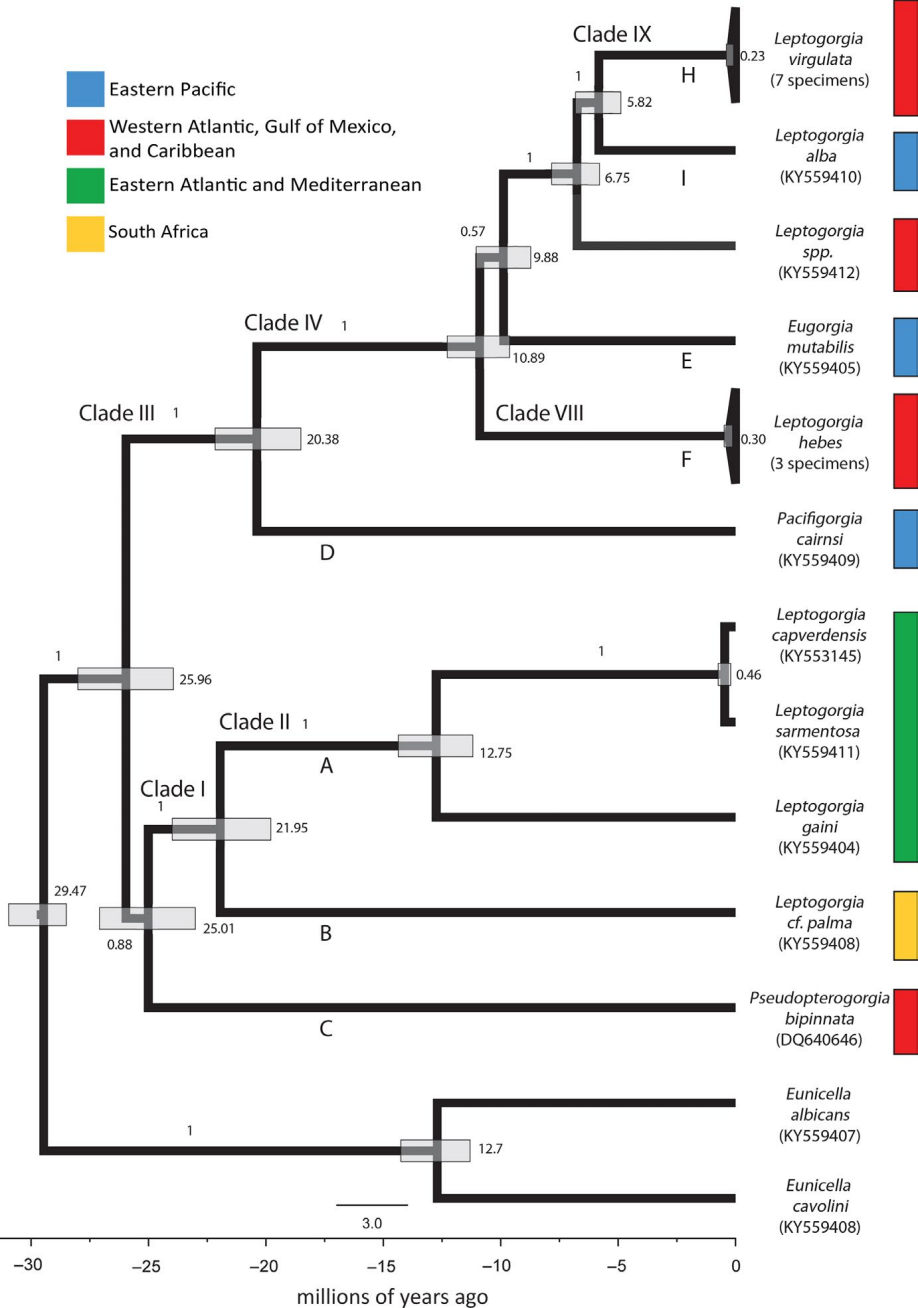
Fossil‐calibrated phylogenetic reconstruction using Bayesian methods, showing divergence times in millions of years ago, indicated by values to the right of the nodes. The scale below the tree is millions of years, and the scale bar is 3.0 million years. Bar labels indicate posterior probability. Color indicates geographic region of species. Branches labeled I–IX as defined by the noncalibrated phylogenetic reconstruction using mitochondrial genomes and those labeled A–I correspond to clades as defined in the phylogenetic reconstruction based on *mtMutS*

The phylogenetic reconstruction based on mitochondrial genomes using fossil‐calibrated coalescent methods as implemented by Bayesian analyses in BEAST (Figure 4) resulted in topology similar to the maximum likelihood (ML) and Bayesian analysis with RaxML and Mr. Bayes, with some key differences. The tree is rooted with two *Eunicella* sp. (outgroup). Emerging from the root are two main clades which diverged from one another 25.96 Ma. Within the first main clade, there are two branches containing a single species each—*P. bipinnata* and *Leptogorgia* cf. *palma* which diverged 25.01 and 21.95 Ma, respectively. Following these two branches is a branch containing *Leptogorgia gaini*, which diverged 12.75 Ma from a sister subclade consisting of *L. sarmentosa* and *L. capverdensis*. *Leptogorgia sarmentosa* and *L. capverdensis* diverged from one another 0.46 Ma. However, this subclade is weakly supported. The grouping of the five aforementioned species is consistent between all three mitogenomic trees, with the exception of *P. bipinnata*. On the ML and Mr. Bayes' Bayesian trees, *P. bipinnata* does not emerge until after the 4 other species—*L*. *cf*. *palma*, *L*. *gaini*, *L. sarmentosa*, and *L. capverdensis*—and it forms a basal branch.

In the second main clade, *P. cairnsi* diverges at 20.38 Ma and forms a basal branch to a subclade containing *L. hebes*, *E. mutabilis*, *Leptogorgia* sp. (KY559412), *L. alba*, and *L. virgulata*. *Leptogorgia hebes* is the first species to diverge from this subclade at 10.89 Ma. Following the *L. hebes* group are two branches containing *E. mutabilis* and *Leptogorgia* sp. (KY559412), diverging at 9.88 and 6.75 Ma, respectively. *Leptogorgia alba* and *L. virgulata* then diverged from one another at 5.82 Ma. In the ML and Mr. Bayes' Bayesian trees, *E. mutabilis* and *L. alba* are sister to one another, but on the BEAST tree *L. alba* is sister to *L. virgulata*.

## 4 DISCUSSION

### 4.1 Mitochondrial MutS phylogeny

The reconstructed *mtMutS* phylogeny uses 68 new *mtMutS* sequences (24 from this study and 44 from GenBank) added to the sequences used in the phylogenetic tree by Poliseno et al. (2017). This new *mtMutS* phylogeny agrees with the phylogeny presented by Poliseno et al. (2017). The *Leptogorgia* species from South Africa form a sister clade to species from the Eastern Atlantic and Mediterranean (Figure 1). The Caribbean clade from Poliseno et al. (2017) is also recovered (clade C, Figure 1). There are several clades with species exclusively from the Eastern Pacific. Most notably, Eastern Pacific clade I is sister to the Western Atlantic and Gulf of Mexico clade H that contains *L. virgulata* and *L. gracilis*. As in Poliseno et al. (2017), the major clades identified (A–I) have species that are exclusive to a particular geographic region. And while all of these clades are strongly supported (except for clade F), relationships between several of these clades is not clear due to low or no statistical support. The South African clade (clade B, Figure 1) contains *L. palma*, formerly known as *Lophogorgia crista*, which is the type species for the *Lophogorgia* genus (Poliseno et al., 2017). Because this South African group is monophyletic and strongly supported, Poliseno et al. (2017) recommend that the genus *Lophogorgia* be resurrected and assigned to this clade. This complicates matters when it comes to other species formerly classified as *Lophogorgia* by Bayer (1961) which are not in the South African clade‐specifically, *L. dichotoma*, *L. capverdensis, L. gaini, Lophogorgia viminalis*, *L. hebes*, *L. punicea*, and *L. violacea*. The former four all belong to the eastern Atlantic clade, while the latter three are Western Atlantic species. Further morphological and genetic analyses of these species in particular will be necessary in order to more accurately classify them and determine whether resurrecting the genus *Lophogorgia* would be appropriate. If the South African clade is recognized as its own genus, whether through the resurrection of *Lophogorgia* or by a new name, it would complicate the taxonomy of the remaining *Leptogorgia* species. The issue is that the type species for the genus *Leptogorgia* is *L. viminalis*, formerly known as *Gorgonia viminalis* (Breedy & Guzman, 2007) is within a monophyletic group with eastern Atlantic‐Mediterranean species, sister to the South African group. Therefore, if the South African group is granted species status, then any species that are not in the sister clade with *L. viminalis* (which are the majority of *Leptogorgia* species) could not be classified as *Leptogorgia* and would have to be renamed. This supports Poliseno et al.'s (2017) call to reclassify almost all *Leptogorgia* species and revise the genus in its entirety, which leaves the case of *L. hebes* all the more ambiguous, as it does not fit in either *Leptogorgia* or *Lophogorgia*. It is likely that new genera need to be defined within this group to resolve these taxonomic issues.

### 4.2 Mitogenomic phylogeny

The complete mitochondrial genomes of 21 gorgonian specimens were examined to elucidate phylogenetic relationships and to test the efficacy of using complete *mt* genome over the single *mtMutS* gene. This is the first study to sequence complete mitochondrial genomes for *L. virgulata* and *L. hebes,* and the resulting mitogenomic phylogeny is in agreement with our *mtMutS* phylogeny and with that of Poliseno et al.'s (2017), albeit with stronger branch support. The tree topology also matches that of the mitogenomic phylogeny presented by Poliseno et al. (2017) while adding *L. hebes* and *L. virgulata* from the Gulf of Mexico as a sister clade to *E. mutabilis* and *L. alba* from the Eastern Pacific. These observations support Poliseno et al.'s conclusions that Western Atlantic gorgonians are more closely related to Eastern Pacific gorgonians than to eastern Atlantic gorgonians (*L. cf. palma*, *L. gaini, L. sarmentosa* and *L. capverdensis*).

### 4.3 Divergence time estimation

This is the first study to place divergence time estimates on complete mitochondrial genomes of *Leptogorgia* species. Poliseno et al. (2017) suggested the first divergence event between Eastern Pacific and Western Atlantic species occurred about 28 Ma (with error bars ranging from 12 to 45 Ma). However, the fossil‐calibrated mitogenomic phylogeny presented in this study suggests that this first split between Eastern Pacific and western Atlantic species occurred later between 11 and 20 Ma (Figure 4), which is within Poliseno et al.'s (2017) lower error range. According to O'Dea et al. (2016), the formation of the Isthmus of Panama was not a singular event, but rather a series of geological events that took place over the course of the last 30 million years. Between 20 and 10 Ma, the Panama Arc island chain began to rise, based on O'Dea et al.'s (2016) estimated rates of Arc uplift. Gene flow by the exchange of gametes and larvae through the CAS was likely high up to10 Ma, while there was still significant seawater exchange between the Atlantic and Pacific oceans (O'Dea et al., 2016). Both, *L. hebes* and *L. virgulata* are adapted to shallow water habitat ranging from 3 to 82 m (Cairns & Bayer, 2009; Williamson et al., 2011). They mature rapidly (<2 years) and are broadcasts spawners, releasing eggs and sperm into the water column (Beasley et al., 2003; Gotelli, 1991). While larval duration in *L. hebes* is not known, it can last up to 20 days in *L. virgulata* (Gotelli, 1991). These characteristics indicate a potential for high dispersal and suggest that gametic and larval connectivity likely occurred between the Pacific and Atlantic oceans through a shallow CAS. Divergence of *Leptogorgia* between these basins likely increased after 10 Ma as seawater exchange became more constricted. Our data suggests that the *L. hebes* speciated at about 11 Ma and it forms the first Western Atlantic clade on the mitogenomic tree. This divergence time coincides with the timing of more restricted water flow between the two basins.

O'Dea et al.'s (2016) uplift data show that after this uplifting period between 20 and 10 Ma, a deepening event occurred between 10 and 6 Ma, in which the Panama Arc began to drop, resulting in greater connectivity between the ocean basins. This span of time is also characterized by shifts in migration rates of both terrestrial and marine fauna, referred to as migration pulses, by Bacon et al. (2015). They specifically highlight a migration shift among marine organisms at around 7.96 Ma, based on their free model migration estimate. The synchrony of submergence of the Panama Arc and a migration event in marine organisms suggest that gene flow could have increased between the Eastern Pacific and Western Atlantic during this time. Following this period of subsidence, at around 6 Ma the Panama Arc began to emerge again and has continued to rise until the present day (O'Dea et al., 2016). Divergences of marine organisms begin to increase at this time, peaking at about 4 million years ago (O'Dea et al., 2016). This timing of events supports the divergence estimate of the Western Atlantic *Leptogorgia* sp. (KY559412) at 6.75 Ma and the divergence of the Western Atlantic *L. virgulata* clade at 5.82 Ma, both diverging from sister clades in the Eastern Pacific. The estimated times of *Leptogorgia* species divergence obtained from this study are concordant with geologic data and historic migration data (Bacon et al., 2015; O'Dea et al., 2016), supporting an initial divergence between Eastern Pacific and Western Atlantic species at about 20–11 Ma with extant lineages arising in each basin in an alternating pattern at 11 (Western Atlantic), 10 (Eastern Pacific), 7 (Western Atlantic), and 6 (Eastern Pacific) Ma (Figure 4).

The divergence times obtained from this study are more recent than those presented by Poliseno et al. (2017) and with lower error estimates (2–4 million‐year range as opposed to a 12–40 million‐year range). This discrepancy is most likely attributed to our use of complete mitochondrial genomes that include fourteen protein‐coding genes and two RNAs instead of a single, partial gene (*mtMutS*). There are numerous studies of multiple taxa showing a pattern of incongruent tree topology between single mitochondrial markers and complete mitochondrial genomes despite the fact that they are the same locus and therefore share the same phylogenetic history (Havird & Santos, 2014; Knaus et al., 2011; Luo et al., 2011; Nadimi et al., 2016; Pacheco et al., 2011; Rohland et al., 2007; Urantowka et al., 2017; Wang et al., 2017; Willerslev et al., 2009). For example, Havird, Santos Scott, and Schierwater, (2014) analyze the performance of single and concatenated sets of mitochondrial genes relative to complete mitochondrial genomes for phylogenetic reconstruction of metazoans. Their findings show that single genes are not able to reproduce the topology of a mitogenomic phylogeny (Havird & Santos, 2014). A similar study, but focusing on birds, showed that single mitochondrial genes resulted in incorrect and contradictory phylogenetic relationships, while the use of complete mitochondrial genomes accurately reflected the species tree (Urantowka et al., 2017). The same pattern has been observed in insects, where individual mitochondrial genes can result in different and contradicting tree topologies, while using the complete mitochondrial genome performs well at various taxonomic levels (Wang et al., 2017). In fungi, the phylogenetic signal differs between single mitochondrial genes, subsets of concatenated mitochondrial genes, and complete mitochondrial genomes, despite all being the same locus (Nadimi et al., 2016). In addition to potentially generating different and contradicting tree topologies, there are numerous examples across widespread taxa on how single mitochondrial genes oftentimes result in poorly supported phylogenetic trees that become fully resolved and well supported when using complete mitochondrial genomes (i.e., Arquez, Colgan, & Castro, 2014; Justice, Weese, & Santos, 2016; Perseke, Golombek, Schlegel, & Struck, 2013; Williams, Foster, & Littlewood, 2014; Yu, Li, Ryder, & Zhang, 2007). Phylogenies in Octocorals present a similar issue; previous research shows that it is difficult to distinguish between species when using the single gene *mtMutS* and that even using a concatenated set of 2–3 different mitochondrial regions only allows to distinguish 70%–80% of morphological species (i.e., Baco & Cairns, 2012; McFadden et al., 2011). The low resolution provided by the use of a single mitochondrial region explains the low support for many clades in the *mtMutS* phylogeny presented in this study and that of Poliseno et al. (2017). Greater resolution and strong support of clades within the Octocorallia is achieved by using complete mitochondrial genomes, as demonstrated in our present study and in previous research (i.e., Figueroa & Baco, 2015, 2014; Kayal et al., 2013; Poliseno et al., 2017).

In addition to incongruent topologies and weakly supported clades, the use of single genes can result in overestimation of calibrated divergence times (Duchêne et al., 2011; McCormack et al., 2011). McCormack et al. (2011) demonstrates that divergence estimation from single mitochondrial genes results in earlier divergence times when compared to the use several markers from the mitochondrial and nuclear genome. They show that the gene tree reconstructed from single mitochondrial markers is not as robust and differs from the species tree reconstructed by using multiple markers from various loci (McCormack et al., 2011). While McCormack et al. (2011) did not examine if complete mitochondrial genomes alone would yield better results, similar to those obtained when using several mitochondrial and nuclear markers, the research by Duchêne et al. (2011) suggests that this might be the case. In their study, Duchêne et al. (2011) compare phylogenetic divergence estimates for cetaceans based on single mitochondrial genes, different combinations of concatenated genes, and complete mitochondrial genomes. Their results show that tree topology from single genes can differ from each other due to different substitution rates and that single gene divergence time estimates consistently resulted in overestimation of divergence times when compared to the use of complete mitochondrial genomes (Duchêne et al., 2011). These results from previous research are congruent with our observations that in octocorals, such as the gorgonians analyzed in our study, the use of complete mitochondrial genomes as opposed to single mitochondrial genes, results in better resolved, well supported, trees that have earlier and more precise divergence time estimates. Since our divergence time estimates are concordant with regional geological events and divergence patterns of other organisms, it supports our hypothesis that the divergence times of Eastern Pacific and Western Atlantic *Leptogorgia* lineages is younger than previously suggested (Poliseno et al., 2017) with the majority of speciation events occurring after 10 Ma when significant seawater exchange between the Pacific and Atlantic Ocean ceased (e.g., Bacon et al., 2015; Montes et al., 2015; O'Dea et al., 2016). However, future work that includes multiple nuclear markers in addition to mitochondrial genomes is necessary to fully test this hypothesis.

## CONFLICT OF INTEREST

The authors declare they have no conflicts of interest.

## AUTHOR CONTRIBUTIONS

DFF conceived the ideas and designed methodology; DH collected the specimens; SS, NJF, and DFF generated the genetic data; SS and NJF analyzed the data; DFF and DH supervised research and analyses; SS and DFF wrote the manuscript; SS, DFF, NJF, and DH contributed to the interpretation of data. All authors contributed critically to the drafts and gave final approval for publication.

### OPEN RESEARCH BADGES

This article has earned an https://openscience.com for making publicly available the digitally‐shareable data necessary to reproduce the reported results. The data is available at https://www.ncbi.nlm.nih.gov/genbank/.

## Data Availability

Mitochondrial genome and *mtMutS* sequences can be accessed online through GenBank (accession numbers listed in Table 4).
